# US trends in the association of suicide ideation/behaviors with marijuana use among adolescents ages 12–17 and differences by gender and race/ethnicity

**DOI:** 10.3389/fpsyt.2022.1057784

**Published:** 2023-01-05

**Authors:** Michael William Flores, Saul Granados, Benjamin Lê Cook

**Affiliations:** ^1^Health Equity Research Lab, Cambridge Health Alliance, Cambridge, MA, United States; ^2^Department of Psychiatry, Harvard Medical School, Boston, MA, United States

**Keywords:** suicidal behavior, suicide attempt, suicide plan, suicide ideation, marijuana use, marijuana use disorder, adolescent

## Abstract

**Background:**

In the US over the past decade, there has been a steady increase in marijuana use rates among adolescents, in part due to marijuana legalization laws. It is unknown whether these greater marijuana use rates are associated with rising rates of adolescent suicide ideation and behaviors (plan and attempt) or whether these associations differ by gender or race/ethnicity.

**Objective:**

To determine whether marijuana use is associated with suicide ideation/behaviors among adolescents and if differences exist by gender and race/ethnicity.

**Materials and methods:**

Using the 2015–2019 National Surveys on Drug Use and Health, we assessed the relationship between marijuana use frequency and suicide ideation/behaviors among adolescents (12–17, *n* = 73,986). We also examined the association with marijuana use disorder (MUD) and assessed differences by gender and race/ethnicity. Marijuana use frequency in the past year was categorized as no use, non-weekly use, and weekly-plus use. We estimated multivariable logistic regression models, adjusting for sociodemographics, health status, common co-occurring behavioral health disorders, and criminal history. For interpretability, regression coefficients were converted into predicted probabilities using predictive margin methods.

**Results:**

In primary analyses, adolescents with non-weekly use and weekly-plus use had higher rates of any suicide ideation, 61.5% (+ 10.4 percentage-points; 95% CI: 7.0–13.8%) and 64.5% (+ 13.4 percentage-points; 95% CI: 9.1–17.7%), relative to no use (51.1%). Non-weekly and weekly-plus use was associated with higher rates of any suicide plan 58.2% (+ 11.8 percentage-points; 95% CI: 7.8–16.0%) and 59.0% (+ 12.6 percentage-points; 95% CI: 6.4–18.9%), and any suicide attempt, 42.0% (+ 11.6 percentage-points; 95% CI: 7.0–16.2%) and 47.3% (+ 16.9 percentage-points; 95% CI: 10.9–22.9%) compared to no use (46.4 and 30.4%, respectively). Similar results were found among adolescents with a MUD (all *p* < 0.05). Positive associations between marijuana use and suicide ideation/behaviors persisted among males and females as well as White, Black, and Latinx adolescents (all *p* < 0.05).

**Conclusion:**

Between 2015 and 2019, suicide ideation/behaviors increased for adolescents that used marijuana. As marijuana is legalized in more states, public health efforts are needed to curb increases in marijuana use among adolescents and to better understand the causal linkages between marijuana use and suicide ideation/behaviors.

## Introduction

Suicide is among the top ten causes of death in the United States (US) and one of the top three leading cause among age groups 10 to 14 and 15 to 24 (1, 2). More than 47,000 individuals died from suicide in 2020 (1, 2). The number of individuals that contemplated suicide, made a suicide plan, or had a suicide attempt were tenfold higher than the number of deaths from suicide (3). The National Comorbidity Study showed that the probability of developing a suicide plan and attempting suicide were 34 and 72% higher for individuals that experienced suicidal ideation, relative to individuals without ideation (4), underlining the importance of deleterious formative suicide behavior that may influence fatal actions.

The rates of marijuana use have increased across the US in the last decade. The legalization of marijuana, both medicinal and recreational, has increased its visibility and availability. As of September 2022, 37 US states and the District of Columbia (DC) allow the medical use of marijuana while 19 US states and the DC have legalized recreational use (5, 6). Among adolescents (individuals between 12 and 17 years of age), 16, 13, and 7% reported using marijuana within their lifetime, within the past year, and within the past month, respectively (7). Older adolescents between 16 and 17 years of age reported the highest rates of use with 31% using marijuana in their lifetime, 25% using in the past year, and 14% using in the past month (7). Adolescents that identified as American Indian/Alaskan Natives had the highest marijuana use rates across lifetime, past year, and past month, followed by Latinx, White, Black, and Asian adolescents (7). The regular use of marijuana among adolescents is associated with negative consequences such as limited scholastic achievement, development of substance use disorders, early onset psychosis, neuropsychological decline, and suicidal behavior (8).

Prior research has found past year cannabis use disorder, daily cannabis use, and non-daily cannabis use was associated with higher prevalence of past-year suicidal ideation, plan, and attempt among adults between 18 and 34 years of age (9). There is also evidence that female adults with cannabis use disorder—both daily and non-daily use—relative to male adults have higher rates of suicidal ideation (9). Less information is known about these associations among adolescents. One longitudinal study using data from Australia and New Zealand assessing the impact of cannabis use prior to age 17 on adult (up to age 30) outcomes showed higher odds of suicide attempts relative to those without any marijuana use in their youth (10). It is unclear if these findings extend to current day experiences of adolescents or if they differ by gender or race/ethnicity. Causal linkages between cannabis use and suicide have not been well-established; these associations are likely partly explained by overlapping risk factors such as depression (9) and psychosis–youth that smoke high-potency marijuana daily are at five times the risk of developing psychosis, a significant risk factor for suicide (11). While unable to elucidate causal pathways, this study documents associations between marijuana use and suicide in order to identify populations at high clinical risk to inform outreach and treatment.

The objective of this descriptive study is to determine whether the frequency of marijuana use and a diagnosis of marijuana use disorder (MUD) are associated with suicide ideation and behaviors (plan and attempt) among adolescents and if differences in these associations exist by gender and race/ethnicity. We hypothesize that: (1) suicide ideation and behavior rates will increase as the frequency of marijuana use increases; (2) suicide ideation and behavior rates will be higher for adolescents diagnosed with a marijuana use disorder than those without a diagnosis; (3) lower rates of suicide ideation and behavior for females compared to males will persist among non-weekly and weekly marijuana users; and (4) suicide ideation and behavior rates will be greater for White individuals than racial/ethnic minority individuals, but these differences will be reduced for minority adolescents with marijuana use because of their greater likelihood of criminal legal involvement related to their marijuana use (12), and other overlapping systemic and interpersonal stressors (13).

## Materials and methods

### Data

We analyzed 2015–2019 data from the National Survey on Drug Use and Health (NSDUH), a cross-sectional survey that captures information on sociodemographics, substance use (e.g., marijuana use), behavioral health disorders, and past-year suicidal ideation, plan, and attempt. The NSDUH collects information from a nationally representative sample of youth and adults that are non-institutionalized US civilians and uses validated diagnostic instruments matching criteria from the Diagnostic and Statistical Manual of Mental Health Disorders (DSM) to identify behavioral health disorders, such as Marijuana Use Disorder (MUD). We pooled 5 years of data to increase the precision of estimates. The interview response rate for the NSDUH ranges from 69.66% in 2015 to 64.92% in 2019 (3, 14). Our analytic sample consisted of adolescents between the ages of 12 and 17 (*n* = 73,986). Analysis of these deidentified, publicly available data was considered exempt from institutional review board review per institutional and federal regulations.

### Dependent variable

Our outcomes of interest were dichotomous variables that reflected any suicidal ideation, plan, or attempt in the previous year. These outcomes are based on responses to three NSDUH questions (3): [1] “Did you think about killing yourself?” [2] “Did you make a plan to kill yourself?” and [3] “Did you make a suicide attempt or try to kill yourself?”

### Independent variables

Our primary independent variable of interest was marijuana use frequency in the previous year. We operationalized marijuana use frequency as a categorical variable reflecting 0 days of marijuana use (hereafter no use), 1 to 51 days of marijuana use (hereafter non-weekly use), and 52 to 365 days of marijuana use (hereafter weekly-plus use) (7). Non-weekly use reflects marijuana use in the previous year that, on average, was less than once a week in a 52-week calendar year. Weekly-plus use reflects marijuana use in the previous year that, on average, was at least once a week in a 52-week calendar year. We estimated regression models, adjusting for factors known to confound the relationship between suicide behavior and marijuana use, including gender (female or male), race/ethnicity [White, Black, Hispanic/Latinx, Asian, Native American and Alaskan Native (NA/AN)], insurance type (private, Medicaid/CHIP, Medicare, other, no insurance), health status (excellent, very good, good, fair/poor), federal poverty level (living in poverty, income up to 2x FPL, income more than 2x FPL), and indicators for major depressive episode (MDE), alcohol use disorder (AUD), nicotine dependence (ND), criminal-legal history, and year (2015, 2016, 2017, 2018, 2019). Our selection of covariates was based on prior research demonstrating a strong conceptual link between the constructs represented by the covariates and suicide ideation/behaviors, and because these covariates could be possible explanatory factors that confound the association between marijuana use and suicide ideation/behaviors of interest. (4, 8, 9, 15, 16).

### Statistical analysis

First, we assessed the characteristics of the analytic sample by marijuana use frequency (all comparisons versus no use) using *t*-test and chi-square tests for continuous and categorical variables, respectively. Second, we plotted unadjusted rates of any suicide ideation, any suicide plan, and any suicide attempt by marijuana use frequency, comparing across groups with chi-square tests. Next, in our primary analysis, we estimated multivariable logistic regression models to assess the association between suicide ideation/behavior and marijuana use frequency, adjusting for covariates described above.

We conducted three sets of secondary analysis. First, to assess whether the association between marijuana use and suicide ideation/behavior changed based on having a clinically recognized marijuana use disorder (MUD) as opposed to frequency of use, we re-estimated regression models with an indicator for MUD. The dichotomous variable, MUD, was operationalized using DSM criteria (3). Second, to identify whether the association between marijuana use frequency and suicide ideation/behaviors differed by gender, we re-estimated regression models with an interaction between marijuana use frequency and an indicator for female gender. We report overall differences by gender, differences within gender by marijuana use frequency, and how the association of marijuana use frequency and suicide varies by gender. Third, we re-estimated regression models looking at the interaction between marijuana use frequency and race/ethnicity to determine if suicide ideation/behavior varied among minoritized groups. We report overall differences by race/ethnicity, differences within race/ethnicity by marijuana use frequency, and how the association of marijuana use frequency and suicide varies by race/ethnicity. In all regression models, we employed predictive margin methods (17) to obtain the between and within group differences and to convert coefficients into percentages for interpretability. We present magnitudes of differences with confidence intervals that allow for comparisons across non-White racial/ethnic groups. All rates and model estimates were weighted to be nationally representative and account for sample design and survey non-response. Survey weights were calculated using general exponential models to account for the five-stage sampling design (including the probability of oversampling), non-response, and extreme weights to ensure survey population was representative of the targeted population distributions, non-institutionalized US civilians (3, 14). Results are considered statistically significant at *p* < 0.05 (2-tailed). Analyses were conducted using Stata release 17 (18).

## Results

Table 1 provides the descriptive characteristics of adolescents by marijuana use frequency. Relative to adolescents with no marijuana use, those with non-weekly use were more likely to be female (54% versus 49%, *p* < 0.001), in school (98 versus 95%, *p* < 0.001), live in households with incomes twice that of the federal poverty level (FPL) (60 versus 56%, *p* < 0.001), of fair/poor health status (5 versus 4%, *p* < 0.01), have an MDE (27 versus 12%, *p* < 0.001), AUD (8 versus 1%, *p* < 0.001), or ND (2 versus 0.3%, *p* < 0.001), and have prior criminal-legal involvement (7 versus 2%, *p* < 0.001). Relative to adolescents with no marijuana use, weekly-plus users were less likely to be female (44 versus 49%, *p* < 0.001) and self-report Asian race (2 versus 7%, *p* < 0.001) than adolescents with no use, and were more likely to live in poverty (23 versus 22%, *p* < 0.05), have fair/poor health status (8 versus 4%, *p* < 0.001), to have a diagnosis of MDE (25 versus 12%, *p* < 0.001), AUD (16 versus 1%, *p* < 0.001), and ND (9 versus 0.3%, *p* < 0.001), and to have prior criminal-legal involvement (18 versus 2%, *p* < 0.001).

**TABLE 1:** Descriptive characteristics of adolescents (12–17) by marijuana use frequency, ^a^National Survey on Drug Use and Health, 2015–2019.

	Marijuana use frequency		
	No use	Non-weekly use	Weekly-plus use
Sample size (N)	59,127	5,447	3,689
	%	%	%
**Sex**			
Female	48.9	53.8***	44.3***
**Race/ethnicity**			
White	54.1	58.0	55.4
Black	14.1	12.8	15.6
Latino	24.7	25.4	25.4
Asian	6.5	3.0***	2.4***
NA/AN	0.6	0.8	1.2***
**Education**			
Attending any type of school	95.2	97.5***	95.6
**Federal poverty level (FPL)**			
Living in poverty	21.8	19.7*	23.3*
Income up to 2x FPL	21.8	20.1**	24.5**
Income more than to 2x FPL	56.4	60.2***	52.2***
**Insurance**			
Private	55.9	56.1	47.7***
Medicaid/CHIP	37.7	37.9	44.3***
Medicare	0.5	0.6	0.6
Other insurance	1.5	1.2	1.7
None	4.4	4.2	5.7**
**Self-rated health**			
Excellent	35.7	29.4***	22.3***
Very good	40.1	42.3*	40.5
Good	20.1	23.1***	29.1***
Fair/poor	4.1	5.3**	8.1***
**Major depressive episode**			
Yes	12.0	27.0***	25.0***
**Alcohol use disorder**			
Yes	0.6	8.3***	16.2***
**Nicotine dependence**			
Yes	0.3	2.1***	9.0***
**Criminal-legal involvement**			
Yes	2.1	6.7***	17.7***
**Year**			
2015	20.0	19.1	21.4
2016	20.2	19.7	17.7
2017	20.0	20.1	19.8
2018	20.0	20.3	19.3
2019	19.8	20.9	21.9

*p < 0.05; **p < 0.01; ***p < 0.001. Comparisons versus no use.

^a^Marijuana use frequency: no use (0 days of marijuana use), non-weekly use (1 to 51 days of marijuana use), weekly-plus use (52 + days of marijuana use).

**FIGURE 1:**
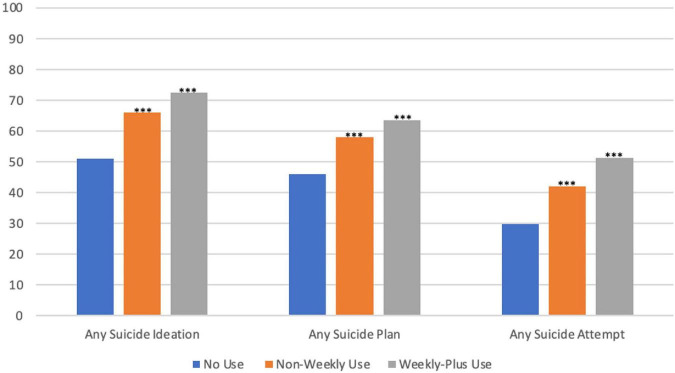
Unadjusted suicide ideation/behavior arates among adolescents by marijuana use frequency and bnational survey on drug use and health, 2015–2019. *p < 0.05; **p < 0.01; and ***p < 0.001; comparisons versus no use. ^a^Behavior includes any suicide plan and any suicide attempt. ^b^Marijuana use frequency: no use (0 days of marijuana use), non-weekly use (1 to 51 days of marijuana use), and weekly-plus use (52+ days of marijuana use).

In unadjusted estimates compared to adolescents with no use (Figure 1), adolescents with non-weekly and weekly-plus marijuana use had higher rates of any suicide ideation (66 and 72% versus 51%, *p* < 0.001), any suicide plan (58 and 64 versus 46%, *p* < 0.001), and any suicide attempt (42 and 51 versus 30%, *p* < 0.001).

In our primary analysis (Table 2), adjusted estimates demonstrated that non-weekly and weekly-plus marijuana use were associated with higher rates of any suicide ideation, 61.5% (+ 10.4 percentage-points; 95% CI: 7.0–13.8%; *p* < 0.001) and 64.5% (+ 13.4 percentage-points; 95% CI: 9.1–17.7%; *p* < 0.001) versus no use (51.1%). Non-weekly and weekly-plus use was also associated with higher rates of any suicide plan, 58.2% (+ 11.8 percentage-points; 95% CI: 7.8–16.0%; *p* < 0.001) and 59.0% (+ 12.6 percentage-points; 95% CI: 6.4–18.9%; *p* < 0.001) versus no use (46.4%), Similarly, non-weekly and weekly-plus marijuana use were associated with higher rates of any suicide attempt, 42.0% (+ 11.6 percentage-points; 95% CI: 7.0–16.2%; *p* < 0.001) and 47.3% (+ 16.9 percentage-points; 95% CI: 10.9–22.9%; *p* < 0.001) versus no use (30.4%).

**TABLE 2:** Adjusted regression estimates of suicide ideation/behavior ^a^by marijuana use frequency ^b^/disorder among adolescents, National Survey on Drug Use and Health, 2015–2019.

	Any suicide ideation			Any suicide plan			Any suicide attempt		
	Base rate	Est.	95% CI	Base rate	Est.	95% CI	Base rate	Est.	95% CI
Marijuana use ^b^(Reference no use)	51.1%	–	–	46.4%			30.4%	–	–
Non-weekly use		10.4%***	7.0–13.8%		11.8%***	7.8–16.0%		11.6%***	7.0–16.2%
Weekly-plus use		13.4%***	9.1–17.7%		12.6%***	6.4–18.9%		16.9%***	10.9–22.9%
Marijuana use disorder (Reference no)	53.1%	–	–	49.1%	–	–	33.5%	–	–
Yes		9.3%**	3.5–15.1%		9.2%**	2.3–16.2%		10.3%**	2.6–18.0%

*p < 0.05; **p < 0.01; ***p < 0.001. Regression model adjusted for race/ethnicity, sex, school attendance, federal poverty level, insurance type, physical health status, MDE, AUD, ND, criminal-legal involvement, and year.

^a^Behavior includes any suicide plan and any suicide attempt.

^b^Marijuana use frequency: no use (0 days of marijuana use), non-weekly use (1 to 51 days of marijuana use), weekly-plus use (52 + days of marijuana use).

Relative to adolescents with no MUD (Table 2), adolescents with a MUD had higher rates of any suicide ideation, plan, and attempt, 62.4% (+ 9.3 percentage-points; 95% CI: 3.5–15.1%; *p* < 0.01), 58.3% (+ 11.6 percentage-points; 95% CI: 2.3–16.2%; *p* < 0.01), and 43.8% (+ 10.3 percentage-points; 95% CI: 2.6–18.0%; *p* < 0.01).

Looking at the overall suicide rates by gender (Table 3), relative to males, females had higher rates of ideation (+ 8.0 percentage-points; 95% CI: 5.1–10.8%; *p* < 0.001), plan (+ 8.6 percentage-points; 95% CI: 4.7–12.5%; *p* < 0.001), and attempt (+ 10.0 percentage-points; 95% CI: 6.5–13.6%; *p* < 0.001). When assessing within gender differences in suicide ideation by marijuana use frequency (Table 3), relative to their no use counterparts, both males and females had higher rates of any suicide ideation if they engaged in non-weekly (+ 9.1 percentage-points; 95% CI: 2.7–15.5%; *p* < 0.01 and + 10.0 percentage-points; 95% CI: 7.3–14.5%; *p* < 0.001, respectively) or weekly-plus use (+ 14.8 percentage-points; 95% CI: 7.7–21.9%; *p* < 0.001 and + 12.8 percentage-points; 95% CI: 7.4–18.1%; *p* < 0.001, respectively). Relative to their no use counterparts, males and females with non-weekly use had higher rates of any suicide plan (+ 13.7 percentage-points; 95% CI: 3.7–23.7%; *p* < 0.01 and + 11.3 percentage-points; 95% CI: 6.8–15.7%; *p* < 0.001, respectively) and any suicide attempt (+ 14.6 percentage-points; 95% CI: 5.8–23.5%; *p* < 0.01 and + 10.8 percentage-points; 95% CI: 5.9–15.7%; *p* < 0.001, respectively). Relative to their no use counterparts, females with weekly-plus use had higher rates of any suicide plan (+ 15.0 percentage-points; 95% CI: 8.9–21.2%; *p* < 0.001), while both male and females had higher rates of any suicide attempt (+ 13.3 percentage-points; 95% CI: 5.0–21.8%; *p* < 0.01 and + 18.4 percentage-points; 95% CI: 11.1–25.8%; *p* < 0.001, respectively). For all suicide outcomes, higher within-group rates associated with marijuana use frequency were not differentially elevated by gender (i.e., the gender*marijuana use frequency interaction terms were not significant).

**TABLE 3:** Adjusted regression estimates of suicide ideation/behavior ^a^by marijuana use frequency ^b^and gender, National Survey on Drug Use and Health, 2015–2019.

Outcome		Overall			Overall difference				95% CI			
Any suicide ideation	Male	48.0%			–				–			
	Female	55.9%			8.0%***				5.1–10.8%			
Any suicide plan	Male	43.4%			–				–			
	Female	52.0%			8.6%***				4.7–12.5%			
Any suicide attempt	Male	26.9%			–				–			
	Female	36.9%			10.0%***				6.5–13.6%			
Outcome		No use	Non-weekly use	Within group difference #1^c^	95% CI	DID #1^d^	95% CI	Weekly-plus use	Within group difference #2^e^	95% CI	DID #2^f^	95% CI
Any suicide ideation	Male	45.3%	54.4%	9.1%**	2.7 to 15.5%	–	–	60.1%	14.8%***	7.7 to 21.9%	–	–
	Female	53.5%	64.4%	10.9%***	7.3 to 14.5%	1.8%	−4.9 to 8.5%	66.3%	12.8%***	7.4 to 18.1%	−2.0%	−10.8 to 6.8%
Any suicide plan	Male	39.8%	53.6%	13.7%**	3.7 to 23.7%	–	–	47.4%	7.6%	−2.9 to 18.0%	–	–
	Female	48.7%	59.9%	11.3%***	6.8 to 15.7%	−2.4%	−13.1 to 8.3%	63.7%	15.0%***	8.9 to 21.2%	7.5%	−2.7 to 17.6%
Any suicide attempt	Male	22.4%	37.0%	14.6%**	5.8 to 23.5%	–	–	35.8%	13.3%**	5.0 to 21.8%	–	–
	Female	33.2%	44.0%	10.8%***	5.9 to 15.7%	−3.8%	−12.9 to 5.3%	51.7%	18.4%***	11.1 to 25.8%	5.1%	−5.3 to 15.4%

*p < 0.05; **p < 0.01; ***p < 0.001. Regression model adjusted for race/ethnicity, school attendance, federal poverty level, insurance type, physical health status, MDE, AUD, ND, criminal-legal involvement, and year.

^a^Behavior includes any suicide plan and any suicide attempt.

^b^Marijuana use frequency: no use (0 days of marijuana use), non-weekly use (1 to 51 days of marijuana use), weekly-plus use (52 + days of marijuana use).

^c^Within group difference #1: Difference between non-weekly use and no use.

^d^DID #1: Difference between within group difference #1 for females and within group difference #1 for males.

^e^Within group difference #2: Difference between weekly-plus use and no use.

*^f^*DID #2: Difference between within group difference #2 for females and within group difference #2 for males.

Looking at the overall suicide rates by race/ethnicity (Table 4), we found no statistically significant differences with the exception that, relative to White adolescents, Latinx had lower rates of ideation (−4.8 percentage-points; 95% CI: −8.3 to −1.4%; *p* < 0.01). When assessing within race/ethnicity differences in suicide ideation by marijuana use frequency (Table 4), relative to their no use counterparts, White, Black, and Latinx adolescents with non-weekly use had higher rates of any suicide ideation (+ 10.9 percentage-points; 95% CI: 6.8–15.1%; *p* < 0.001, + 12.9 percentage-points; 95% CI: 2.5–23.3%; *p* < 0.05, and + 8.6 percentage-points; 95% CI: 0.9–16.2%; *p* < 0.05, respectively) any suicide plan (+ 10.9 percentage-points; 95% CI: 5.6–16.0%; *p* < 0.001, + 20.5 percentage-points; 95% CI: 9.1–32.0%; *p* < 0.001, and + 10.7 percentage-points; 95% CI: 2.0–19.4%; *p* < 0.05, respectively). Relative to their no use counterparts, White, Black, and NA/AN adolescents with non-weekly use had higher rates of any suicide attempt (+ 12.0 percentage-points; 95% CI: 6.0–18.0%; *p* < 0.001, + 19.6 percentage-points; 95% CI: 6.8–32.4%; *p* < 0.01, and + 29.9 percentage-points; 95% CI: 1.7–58.1%; *p* < 0.05, respectively). Relative to their no use counterparts, White and Latinx adolescents with weekly-plus use had higher rates of any suicide ideation (+ 14.9 percentage-points; 95% CI: 9.3–20.5%; *p* < 0.001, + 14.3 percentage-points; 95% CI: 5.4–23.3%; *p* < 0.01), White had higher rates of any suicide plan (+ 16.5 percentage-points; 95% CI: 9.7–23.3%; *p* < 0.001), and White and Latinx had higher rates of any suicide attempt (+ 19.1 percentage-points; 95% CI: 11.8–26.4%; *p* < 0.001, + 15.3 percentage-points; 95% CI: 1.3–29.3%; *p* < 0.05). For all suicide outcomes, higher within-group rates associated with marijuana use frequency were not differentially elevated by race/ethnic group (i.e., the race/ethnicity*marijuana use interaction terms were not significant).

**TABLE 4:** Adjusted regression estimates of suicide ideation/behavior ^a^by marijuana use frequency ^b^and race/ethnicity, National Survey on Drug Use and Health, 2015–2019.

Outcome		Overall			Overall difference				95% CI			
Any suicide ideation	White	54.4%			–				–			
	Black	54.7%			0.3%				−3.6 to 4.3			
	Latinx	49.5%			−4.8%**				−8.3 to −1.4			
	Asian	59.5%			5.1%				−0.5 to 10.8			
	NA/AN	56.2%			1.8%				−7.1 to 10.6			
Any suicide plan	White	49.6%			–				–			
	Black	45.9%			−3.7%				−8.6 to 1.3			
	Latinx	51.7%			2.1%				−1.5 to 5.7			
	Asian	49.1%			−0.5%				−8.2 to 7.2			
	NA/AN	46.5%			−3.0%				−20.3 to 14.3			
Any suicide attempt	White	33.5%			–				–			
	Black	34.9%			1.4%				−3.2 to 6.0			
	Latinx	35.9%			2.4%				−1.4 to 6.3			
	Asian	32.0%			−1.5%				−8.7 to 5.7			
	NA/AN	34.4%			0.9%				−13.1 to 15.0			
Outcome		No use	Non-weekly use	Within group difference #1^c^	95% CI	DID #1^d^	95% CI	Weekly-plus use	Within group difference #2^e^	95% CI	DID #2^f^	95% CI
Any suicide ideation	White	51.8%	62.7%	10.9%***	6.8 to 15.1%	–	–	66.7%	14.9%***	9.3 to 20.5%	–	–
	Black	52.4%	65.2%	12.9%*	2.5 to 23.3%	2.0%	−9.4 to 13.3%	59.1%	6.8%	−5.1 to 18.7%	−8.2%	−20.3 to 4.0%
	Latinx	47.2%	55.7%	8.6%*	0.9 to 16.2%	−2.4%	−11.0 to 6.3%	61.5%	14.3%**	5.4 to 23.3%	−0.6%	−11.1 to 9.9%
	Asian	59.5%	64.8%	5.4%	−16.1 to 26.9%	−5.6%	−27.5 to 16.4%	51.4%	−8.0%	−37.6 to 21.5%	−23.0%	−53.6 to 7.6%
	NA/AN	52.2%	73.4%	21.2%	−9.9 to 52.4%	10.3%	−20.2 to 40.8%	58.2%	6.0%	−23.3 to 35.3%	−8.9%	−39.2 to 21.4%
Any suicide plan	White	46.1%	56.9%	10.9%***	5.6 to 16.0%	–	–	62.6%	16.5%***	9.7 to 23.3%	–	–
	Black	41.4%	61.9%	20.5%***	9.1 to 32.0%	9.7%	−2.7 to 22.1%	50.8%	9.5%	−4.4 to 23.4%	−7.0%	−22.9 to 8.9%
	Latinx	49.1%	59.9%	10.7%*	2.0 to 19.4%	−0.1%	−10.0 to 9.9%	54.0%	4.9%	−8.0 to 17.8%	−11.6%	−24.6 to 1.4%
	Asian	47.5%	59.2%	11.8%	−9.5 to 33.1%	1.0%	−21.6 to 23.5%	47.9%	0.5%	−40.2 to 41.2%	−16.0%	−57.7 to 25.7%
	NA/AN	42.3%	55.0%	12.6%	−20.8 to 46.0%	1.8%	−31.7 to 35.3%	54.5%	12.2%	−21.6 to 45.9%	−4.3%	−36.4 to 27.8%
Any suicide attempt	White	29.4%	41.4%	12.0%***	6.0 to 18.0%	–	–	48.5%	19.1%***	11.8 to 26.4%	–	–
	Black	30.7%	50.3%	19.6%**	6.8 to 32.4%	7.6%	−6.1 to 21.4%	38.4%	7.7%	−7.4 to 22.9%	−11.4%	−28.3 to 5.5%
	Latinx	32.9%	39.7%	6.8%	−2.1 to 15.8%	−5.1%	−15.5 to 5.3%	48.2%	15.3%*	1.3 to 29.3%	−3.8%	−19.2 to 11.6%
	Asian	30.0%	42.2%	12.3%	−10.1 to 34.6%	0.3%	−23.1 to 23.7%	31.0%	1.1%	−29.1 to 31.2%	−18.0%	−48.0 to 11.9%
	NA/AN	26.1%	56.0%	29.9%*	1.7 to 58.1%	17.9%	−11.0 to 46.7%	52.7%	26.5%	−6.7 to 59.8%	7.4%	−24.1 to 39.0%

*p < 0.05; **p < 0.01; ***p < 0.001. Regression model adjusted for race/ethnicity, gender, school attendance, federal poverty level, insurance type, physical health status, MDE, AUD, ND, criminal-legal involvement, and year.

^a^Behavior includes any suicide plan and any suicide attempt.

^b^Marijuana use in the past year: no use (0 days of marijuana use), non-weekly use (1 to 51 days of marijuana use), weekly-plus use (52 + days of marijuana use).

^c^Within group difference #1: Difference between non-weekly use and no use.

^d^DID #1: Difference between within group difference #1 for racial/ethnic minority and within group difference #1 for white.

^e^Within group difference #2: Difference between weekly-plus use and no use.

^f^DID #2: Difference between within group difference #2 for racial/ethnic minority and within group difference #2 for white.

## Discussion

Using nationally representative data, our findings demonstrated that past-year marijuana use is a significant risk factor for suicide ideation/behavior among adolescents. This finding was consistent among males and females, as well as adolescents identifying as White, Black, Latinx, and NA/AN. We also found that rates of suicide ideation/behavior increased as the frequency (number of days) of marijuana use increased. While prior literature has found gender, race/ethnicity, and marijuana use to be independent factors associated with suicide ideation/behavior among adolescents (19), our study is one of the first to use a nationally representative sample of adolescents to examine associations between suicide ideation/behavior and marijuana use and how these associations differ by gender and race/ethnicity.

Marijuana use, which we found to be associated with higher rates of suicide ideation/behavior, is influenced by a multitude of factors, including supply side factors such as availability, price, and potency. Marijuana has become more widely available *via* recreational use legislation. As of September 2022, 19 states and the District of Columbia have enacted measures that allow non-medical marijuana use (6). Increased marijuana availability may result in adolescents initiating or increasing their recreational use. In an analysis of a national, annually administrated cross-sectional survey, Cerda and colleagues found marijuana prevalence rates among eighth and tenth graders in the state of Washington increased when comparing marijuana use rates before and after legalization (20). The state saw an increase in the prevalence of habitual marijuana users and a decrease in the prevalence of non-users. With legalization, the stigma of marijuana use may dissipate and elevate the social acceptability, which can lower the perceived risks associated with marijuana use (20, 21). As such, adolescents living in states with legalized recreational marijuana legislation for adults may initiate or increase their use without fully considering consequences (e.g., elevated risk of psychosis and impacts of brain development associated with adolescent marijuana use).

The decreasing price of marijuana may also contribute to increasing use among adolescents and help to further explain our research findings. Marijuana prices in the black market have decreased in the advent of recreational marijuana legalization (22) potentially becoming more accessible to adolescents (only adults aged ≥ 21 are able to legally purchase recreational marijuana). Before legalization, black market marijuana could be sold at a premium to offset costs from criminal-legal involvement (20, 23). Over time, the saturation of recreational marijuana vendors and increased efficiency and maturation of the marijuana industry may also drive down the price of legal marijuana diverted to adolescents (23). While no studies, to our knowledge, have assessed recreational marijuana diversion among adolescents, we can look to the medical marijuana literature as a reference. One study found 74% of adolescents (14–18) had used someone else’s medical marijuana in the past year (24). The rate of diverted medical marijuana among adolescents was comparable to that of adults (25). Future research should focus on adolescents and assess the impact of recreational marijuana diversion and elucidate the extent to which black market marijuana pricing influences initiation and use frequency.

As markets, both legal and illegal, compete for customers, there has been a proliferation of potent marijuana products, which may have severe consequences for adolescents. The levels of Δ9-tetrahydrocannabinol (THC), the content that gives marijuana its euphoric effects, have significantly increased over the past several decades (26). THC is associated with acute increases in heart rate, various types of arrhythmias, coronary vasospasm, and acute myocardial infarction (26). As marijuana potency has increased, there may be a parallel escalation in mental health-related events. Work by Di Forti and colleagues (11) found daily adult (18–64) use of high potency marijuana, defined as THC ≥ 10%, was associated with five-times the odds of having a psychotic disorder, relative to no use. Researchers determined that eliminating high potency marijuana would contribute to a 12% decrease in the number of first-episode psychosis cases. This is of critical importance as psychosis is a predictor of suicidal behavior. A systematic review and meta-analysis of 10 general population cohort studies (27) found individuals with a psychotic experience had higher odds of suicidal ideation, plan, attempt, and suicidal death. To date, there is little to no guidance or regulation on marijuana potency. Only two US states have placed limits on THC potency (28) despite the empirical evidence highlighting potential adverse effects. State policymakers, public health officials, and researchers should work together to ensure the development of regulations that are evidence-based and take into account adolescent health.

Our finding of higher suicide ideation/behavior for males and females that use marijuana, relative to no use are similar to findings among young adults between the ages of 18–34 (9). This is critical as prior research has shown a history of suicide behavior is a strong predictor for suicide death (15) and concerning national trends showing increasing suicide deaths for both males and females between 2000 and 2020 (29).

We did not find between gender differences in any suicide outcomes regardless of marijuana use frequency. While epidemiological studies have shown high school aged females have higher rates of suicide ideation, plan, and attempt compared to their male counterparts (7), our findings suggest that this difference between gender groups may be moderated by marijuana use. Youth between the ages of 12–17 have comparable lifetime, past-year, and past month rates of marijuana use (30). The limited available literature is mixed on how marijuana affects gender groups differentially. For example, some studies have found marijuana use increases the risk of first-episode psychosis (a significant risk factor for suicide) among males than females (31, 32), while other research have found no gender differences in the development of psychosis among marijuana users (33, 34). There is a need for longitudinal studies to take into consideration the heterogeneity among gender groups when evaluating the causal relationship between marijuana use and suicide ideation/behavior. Additionally, prevention efforts should target both male and female adolescents and focus on early screening and treatment for marijuana use.

Prior descriptive epidemiological studies (3, 35) among high school adolescents have shown NA/AN and Asians have the highest rates of suicidal ideation followed by White and Black (tied with Latinx). NA/AN adolescents also had the highest rates of making a suicide plan followed by Asian (tied with White) and Black (tied with Latinx). Attempting suicide was also highest among NA/AN adolescents followed by Black, Latinx, and Asian (tied with White) adolescents. Our findings are also suggestive of concern for high rates of suicide behavior among NA/AN adolescents as we identified that NA/AN adolescents with non-weekly marijuana use had higher rates of suicide attempts than their White counterparts. While the size of the sample of NA/AN adolescents is a limitation, this significant result adds to the scant literature in this area. Future studies assessing associations between marijuana use and suicide among NA/AN adolescents are needed.

In our literature search, there was little to no research on how the associations between marijuana use and suicidal ideation/behavior vary by race/ethnicity. One study using 2004 National Violent Death Reporting System data for 13 US states found no differences in suicide rates among racial/ethnic groups by marijuana use (36). In our study, for all racial/ethnic groups, we identified an increased risk of suicide ideation/behavior with greater frequency of marijuana use, and that this increase in risk did not vary significantly by race/ethnicity. Our hypothesis was that the suicide ideation/behavior rates of Black and Latinx adolescents with marijuana use would converge to the higher rates of White adolescents given the additional criminal legal involvement and covarying structural stressors prevalent among Black and Latinx adolescents marijuana users. More research related to the intersection of structural racism, marijuana use, and suicide, [e.g., the criminalization of marijuana use among minoritized adolescents (12)], and resilience to other structural stressors regularly faced by these minoritized youth is needed.

Our findings should be considered amidst several limitations. First, the NSDUH is cross-sectional and precludes our ability to make causal inferences. Nonetheless, our study illustrates significant associations between marijuana use and suicide ideation/behavior among a nationally representative sample of adolescents. Second, data are based on survey respondent’s self-reports and dependent on their recall. To increase the accuracy of survey responses, answers to sensitive questions are collected using Audio-Computer Assisted Self-Interview methods, where respondents listen to prerecorded questions using headphones and enter their responses directly into a NSDUH laptop computer without NSDUH interviewers knowing their answers (37). Third, the survey population is limited to non-institutionalized US civilians and excludes individuals that are homeless, incarcerated, or in residential treatment. Consequently, our estimates may be conservative as the aforementioned groups have been shown to have higher rates of marijuana use (38) and suicidality (39). Fourth, we used a measure of marijuana use in the previous year that does not capture potency (e.g., THC concentration) or manner of ingestion (e.g., vaporizer). Fifth, the NSDUH does not capture suicidal preparation, intent, or an individual’s impulsivity, which are critical components of suicide behavior. Finally, although we adjusted for many variables that may confound the relationship between marijuana use and suicide ideation/behaviors, due to survey data limitations, there were no available variables that describe traumatic experiences, exposure to violence, housing insecurity, and other measures of disenfranchisement known to be associated with suicide ideation/behaviors; this was a limitation to our explanatory model and may have resulted in omitted-variable bias.

We conclude that greater frequency of marijuana use and a marijuana use disorder diagnosis among adolescents were both associated with higher rates of suicidal ideation/behavior among a nationally representative sample of US adolescents. As marijuana has become more widely available *via* legalized adult recreational use legislation, public health efforts are needed to curb growing illicit use among adolescents. Marijuana has many medicinal benefits such as reducing anxiety, minimizing inflammation, and increasing pain relief. As such, marijuana should continue being responsibly used among those with physician-approved need. In terms of recreational use, there should be policies that are rooted in anti-racist principles that enable users from all racial/ethnic backgrounds to freely engage in a legal activity without persecution. Similar to alcohol and tobacco, adolescents should be readily informed of the potential consequences of marijuana use. There is a need to educate and enforce safe and responsible marijuana consumption by legal age users. Moreover, the pathways leading increased marijuana use to higher rates of suicide ideation/behavior are not well-known and should be investigated in mechanistic analyses to inform effective interventions.

## Data availability statement

Publicly available datasets were analyzed in this study. This data can be found here: https://www.datafiles.samhsa.gov/.

## Author contributions

MF, SG, and BC contributed to the conception/design, acquisition, analysis, interpretation of data for this study, and drafting and critically revising of the manuscript. All authors agreed to be accountable for all aspects of the work and approved the publication of the manuscript.
